# The Present States of Pituitrin in Obstetrics

**Published:** 1914-05

**Authors:** James Robert McCord

**Affiliations:** Atlanta, Ga.


### THE PRESENT STATES OF PITUITRIM IN OBSTETRICS.

By James Robert McCord, M. D., Atlanta, Ga.

Pituitrin, though a comparative recent addition to our obstetrical bag, is so widely used, and as I believe, in many instances, so wrongly used, that the writer has endeavored to briefly summarize from the literature and his own practical observations, some of the indications, coutra-indications and effects of Pituitrin when administered hypodermically to pregnant or pavtuvient women.

Humpstone has shown that only in occassional cases, will large doses (4 cc.) of pituitrin, repeated every day for 3 or 4 days, induce labor at term or beyond.

In 3 or 4 cases of my own I was not able, in any of them, to start labor.

It has been proven, and fortunately so, that very large doses of the drug will not, in any case, produce abortion.

I do not believe that pituitrin is ever indicated in a case of normal labor, assuming that simple inertia in the second stage is abnormal. The time element, is, as a rule, not a very important thing in the management of a case of normal labor. The administration of pituitrin to hasten labor for the comfort or convenience of the obstetrician, is no more justifiable than the application of forceps for the same purpose. Unfortunately for the women concerned, both are often done.

The action of pituitrin on the uterus is the development, 5 to 10 minutes after administration, of rhythmic (as a rule, but not always so) uterine contractions, with wide variations of severity. The contractions may be mild or so severe as to be uncontrollable, and absolutely clonic in character.

As a rule pituitrin acts better with multipara than primipara. Why this is true I do not know.

Drs. DeLee of Chicago and McPherson of the New York Lying-In Hospital, both state that post partum hemorrhage is considerably more frequent after the administration of Pituitrin. This has been my experience in several cases, and I have heard of on or two recently from other men. In fact, there are numerous such reports that can be found in obstetrical literature. I have wondered if the severe uterine contractions, caused by the drug, and the resistance offered to these contractions by the child, did not cause a partial exhaustion or paralysis of the uterus—and that after the expulsion of the child and placenta, that this paralysis prevented a firm contraction of the uterus.

I do not think that Pituitrin should be given at all during the first stage of labor. If given at this time certain conditions must be present, or the administration of the drug is a hazardous procedure.

If the membranes are unruptured the cervix should be at least 3 fingers dilated and well thinned out. If the membranes are ruptured, the cervix should be at least four fingers dilated, and were obliterated. To give pituitrin before the cervix is thinned is a dangerous procedure. I have had one case where multiple incisions of the cervix were necessary because the drug wasgiven before dilatation had gone far enough.

The drug is of value in the second stage of labor, when there is an uterine inertia, and no disproportion between the head and the pelvis, and no abnormal rotation of the presenting part. I do not think that a tired uterus, struggling to rotate and occiput posterior, should ever be given Pituitrin, that nature will be better aided by the hypodermic administration of ¼ to ½ grain of morphia, or full doses of chloral and bromide per rectum.

I think that I have several times avoided the application medium and low forceps by the judicious use of Pituitrin in the second stage.

The drug should never be used to try and force the engagement of a foetal head, certainly not in a primipara, and I question the wisdom of it even in multipara, with a previous history of easy labors. Competent obstetricians say that it can be used in minor degrees of pelvic contraction, to add the small amount of force necessary to push the head through. I do not agree, because it is so hard to tell how much force will be necessary, and again because of the difficulty in estimating the size of the foetal head in utero.

The best of our abstetricians often are entirely wrong in their estimations of the size of a head even when engagement has occurred. When the drug is given for uterine inertia, 1 cc. is the preferred dosage, though as much as 4 cc. have been administered with no evident ill effects.

In some cases the contractions soon stop, and the dose should be immediately repeated. Occassionally we find a case where the drug has absolutely no effect at all.

Care should be exercised to thoroughly empty the rectum and bladder, before giving Pituitrin.

Pituitrin is in my opinion the most valuable drug we posess to combat post portum hemorrhage caused by uterine relaxation. It is beter to use it in combination with some form of Ergot, so that the quick acting Pituirin, may be reinforced by the more prologned action of ergot. I can not help but add here, that uterine tamponade, is in my opinion the most national way to treat post portum hemorrhage, and not to delay doing this until your patient is exsanguinated. I have found nothing in the literature where Pituitrin has been used to aid in the expulsion of the placenta, though I know two or three men that use it, not infrequently for this purpose. I can see no valid reason for its use. A true adherent placenta is an exceedingly rare occurrence, and I think if the placenta were really adherent that Pituitrin would only complicate the condition. Aplacenta left alone long enough after labor, can always be expressed by Crede's method properly applied.

In cases of sub involution of the uterus following labor, where about the fifth or sixth day post portum, there is more or less uterine hemorrhage, Pituitrin works wonders. I have seen several cases of hemorrhage from subinvolution, in which rest, ergot, etc., was tried, for one week, two weeks, three weeks with no results, immediately stopped with the first dose of Pituitrin.

When given in these cases it should be given in 1cc. doses, once or twice a day as needed.

Among the accidents that have been reported from the use of Pituitrin are:

Post Portum hemorrhage
Rupture of the uterus.
Premature expulsion of the placenta.
Tetany of the uterus.
Recto cervical festula.
Toxic convulsions in the child.
Impairment, slowing of heart and asphyxia.
In version of the uterus.

In conclusion, I believe that the administration of the drug should be confined to the following indications.

1. Uterine inertia, second stage, normal presentation.
2. Past Portum hemorrhage, due to anatonic condition of the uterus.
3. Subinvolution of the uterus.

